# Preserved Mandibular Bone Microarchitecture Following Ovariectomy-induced Osteoporosis is Associated with a Specific Fatty Acid Composition in the Rat

**DOI:** 10.1007/s00223-026-01543-5

**Published:** 2026-05-07

**Authors:** Maxime Bedez, Nicolas Bertheaume, Jérôme Delattre, Cécile Olejnik, Alexandrine During

**Affiliations:** Univ. Lille, CHU Lille, Univ. Littoral Côted’Opale, ULR 4490 - MABLab- Adiposité Médullaire et Os, F-59000 Lille, France

## Introduction

Osteoporosis is a progressive metabolic disease characterized by reduced bone mass and disrupted microarchitecture, increasing fracture risk. It primarily affects postmenopausal women, affecting 35.3% of women [1]. Traditional assessments using bone mineral density (BMD) alone fail to predict fracture risk among individuals with similar values [2], emphasizing the importance of bone quality, including architectural and compositional characteristics.

Bone marrow adipocytes (BMAds) are major cells of bone marrow (BM), forming the bone marrow adipose tissue (BMAT), comprising approximately 70% of marrow volume [3]. BMAT volume and lipid composition vary considerably among bones and individuals [4, 5]. Premenopausal women have lower BMAT levels compared to men, increasing significantly after menopause, correlating with osteopenia and osteoporosis [6–8]. BMAT may influence bone quality and metabolism. Previous work from our team on the rat tibia demonstrated that adipose tissue correlated negatively with bone volume and positively with a highly organized mineral phase [9].

BM lipid composition provides insight beyond energy storage, influencing bone fragility and inflammation. Recent studies report specific lipid profile alterations linked to bone diseases, highlighting pro-inflammatory and anti-inflammatory fatty acids (FAs) in osteoarthritis and fractures [10, 11]. Arachidonic acid (AA, 20:4 *n* − 6) occupies a paradoxical position: as a precursor of prostaglandins and thromboxanes, it sustains pro-inflammatory signaling, yet higher dietary intake has been associated with reduced hip fracture risk [12], and osteoporotic patients display lower circulating levels [13]. This dual role makes 20:4 *n* − 6 a key lipid to monitor in bone health. The enzyme Δ9 desaturase (stearoyl-CoA desaturase) converts saturated FAs (SFAs) into monounsaturated FAs (MUFAs): stearic acid (18:0) to oleic acid (18:1 *n* − 9) and palmitic (16:0) to palmitoleic acid (16:1 *n* − 7). Elevated desaturation indices have been linked to estrogen-deficiency bone loss in the ovariectomized (OVX) rat model [14] and to higher fracture risk in men [15]. In addition, a recent longitudinal study from our team demonstrated that the decrease in cortical bone thickness and the increase in cortical porosity were strongly associated with both 18:1/18:0 ratio and 20:4 *n* − 6 catabolism, in femurs of aged and OVX rats [16]. These alterations highlight the influence of lipid metabolism on bone remodeling and mineralization and suggest that Δ9 desaturase indices and 20:4 *n* − 6 metabolism are candidate pathways mediating lipid-driven skeletal fragility. However, the underlying mechanisms driving these associations require further investigation.

Mandibular lipid composition remains underexplored compared to long bones. Studies reveal the mandible’s resistance to estrogen deficiency, maintaining bone density and mechanical properties [17]. Mandibular BMAT is significantly lower than in long bones across various species, including primates [18], although data in humans remain currently unavailable.

Here, we investigate the mandibular lipid profile to define its distinct biochemical signature, providing new insights into its unique metabolism and inherent resistance to osteoporotic degradation.

In female Sprague-Dawley rats, we investigated whether estrogen deficiency induced by ovariectomy, compared to sham-operated (SHAM) controls, affects the mandibular fatty acid profile, including individual proportions and ratios in both MT and BM. A secondary question addressed the site effect, namely whether mandibular and tibial lipid profiles differ under both SHAM and OVX conditions in MT.

## Methods

### Material

Lipid standards were purchased from Sigma-Aldrich (72332-1G; Saint-Quentin-Fallavier, France). For high performance liquid chromatography (HPLC) analyses, methanol, dichloromethane, and acetonitrile of HPLC grade were purchased from Sigma-Aldrich.

### Animals and Bone Collection

This study was designed and reported in accordance with the ARRIVE 2.0 guidelines. The completed checklist with page references is provided as Supplementary Material. 5.5-month-old female Sprague–Dawley rats (Charles River, Saint-Germain-Nuelles, France) underwent sham surgery (SHAM group, *n* = 10) or ovariectomy (OVX group, *n* = 10) performed by the provider, and were then transferred at six months to the conventional animal facility of the University Hospital Experimental Research Department (DHURE, Lille University, France, authorization B 59–35010). Rats were free of known viral, bacterial and parasitic pathogens (specific-pathogen-free status). At delivery, they had a body weight of 334 g ± 33 g. After transferring to DHURE facility and routine housing, animals were maintained for 5 months post-surgery, reaching 11 months of age at the study endpoint. Animals (*n* = 2/cage) were housed in type 4 cages filled with Lignocel^®^^,^ enriched with cotton, horizontal tubes for climbing and wood sticks for chewing, under controlled conditions (22 °C ± 2 °C, 12 h light/dark, lights on 7 a.m.–7 p.m.). They were fed standard pellet diet (AIN−93G^®^, Bio-Serv) and water *ad libitum*. All animal experiments were carried out under the approval of the French Ministry of Research (APAFIS no. 2016040110346581), as part of an ancillary protocol derived from During et al. (ongoing), in accordance with Directive 2010/63/EU. Animals meeting predefined clinical or behavioral exclusion criteria were removed from the study, regardless of their assigned group. Beyond weight monitoring, no treatments or procedures were performed post-surgery to prevent confounding experimental biases. As all outcomes were assessed post-mortem, masked assessment was not required. At 5 months post-surgery, rats were euthanized by decapitation under anesthesia with isoflurane. Bone samples (mandibles and tibiae) were quickly harvested, cleaned from soft tissues, and frozen at − 80 °C. In this study, only right hemimandibles were analyzed, whereas tibiae from both the right and left sides were pooled prior to analysis.

### Microarchitecture Analysis by Microtomography

**Fig. 1:**
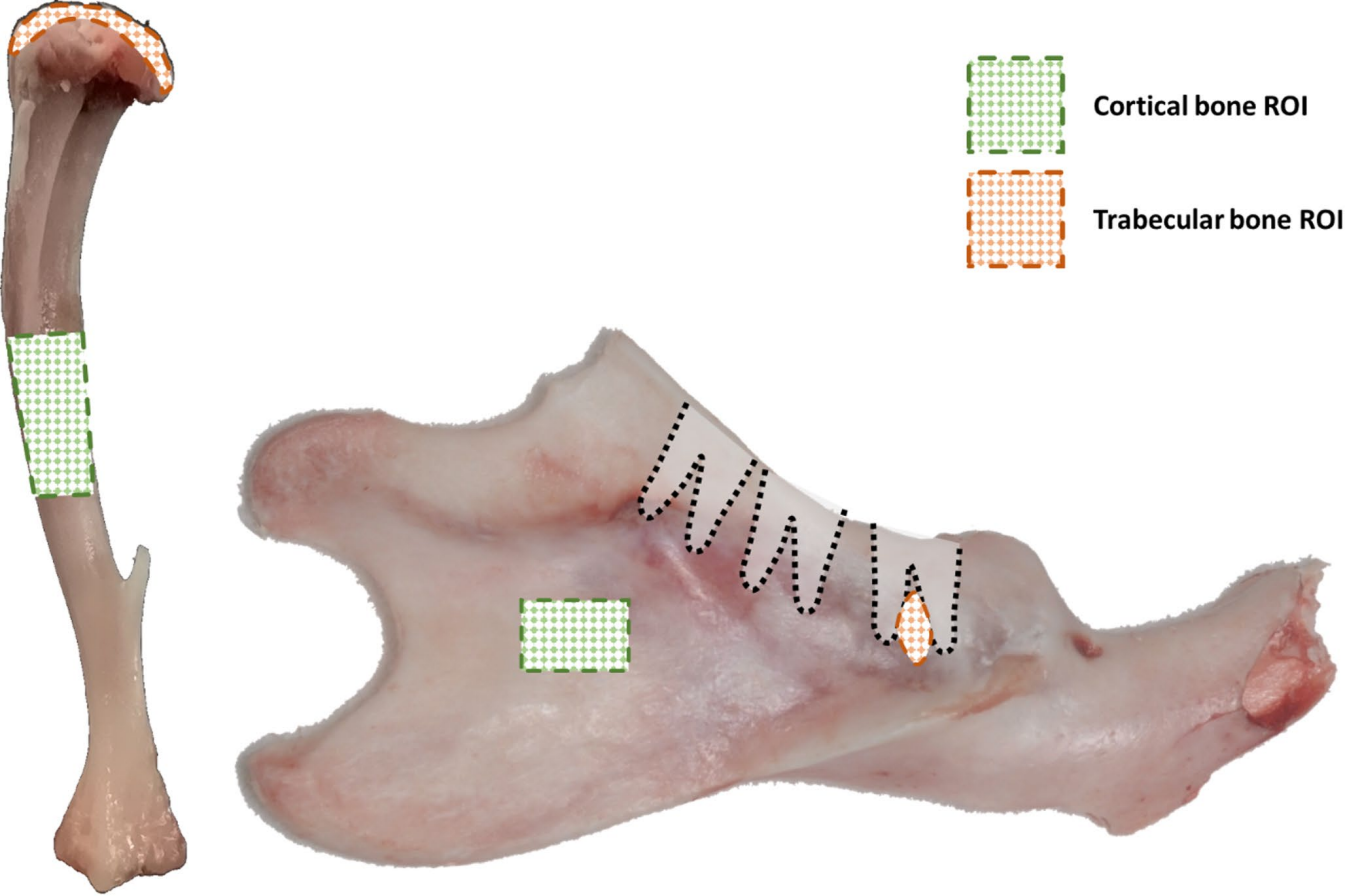
Regions of interest in the tibia (left) and the mandible (right) for microarchitecture analysis

To confirm the osteoporotic status of the animals, tibial microarchitecture was assessed using a Skyscan 1172 micro-computed tomography (µCT) scanner (Bruker, Kontich, Belgium). Throughout this process, the mandibular bones were kept at subzero temperatures to preserve lipid composition for subsequent analysis. To enable cryo-µCT scanning, we designed a custom thermal reservoir, printed in Clear V4^®^ resin using a Formlabs SLA printer (Formlabs, Somerville, MA, USA). This thermal reservoir was fitted into the Skyscan system and filled with pre-cooled isopropanol (− 80 °C), while the bone samples were embedded in powdered dry ice (− 78.5 °C) – see Supplementary Materials, Fig. S2 for additional details. Scan settings were as follows. For the mandibles: powdered dry ice as the scanning medium, 100 kV, 100 µA, Al-Cu filter, 1100 ms integration time, frame averaging 1, 0.9° rotation step over 180°, and an isotropic voxel size of 20 μm. For the tibiae: no medium, 90 kV, 110 µA, Al-Cu filter, 1250 ms integration time, frame averaging 3, 0.3° rotation step over 180°, and an isotropic voxel size of 4 μm. The differences between the scan settings were made to maintain a reasonable timeframe for the acquisition of the mandibular bone, to keep it cooled with the thermal reservoir (around 40 min). Reconstruction was performed using NRecon 1.7.4.6 (Bruker, Kontich, Belgium). For the mandibles, parameters included 36% beam hardening correction, ring artifact correction level 4, smoothing 2, and a CS-to-image conversion range of 0 to 0.024. For the tibiae, reconstruction used a 50% beam hardening correction, ring artifact correction level 4, smoothing 1, and a CS-to-image conversion range of 0 to 0.076. The regions of interest (ROIs) were drawn using Avizo 2019.4 (FEI Visualization Sciences Group, Houston, USA) for the cortical bone, and using CTAn 1.19 (Bruker, Kontich, Belgium) for the trabecular bone. The ROI was determined at the secondary ossification center of the proximal epiphysis for the tibial trabecular bone, at the center of the diaphysis for the tibial cortical bone, at the space between the roots of the first molar for the mandibular trabecular bone, and behind the roots of the third molar, on the buccal side, for the mandibular cortical bone (Fig. 1, see Supplementary Materials, Figure S1 for illustrated ROIs). In these ROIs, a one-level Otsu’s thresholding method was applied to distinguish MT from the BM compartment. Structural parameters were quantified using CTAn software. For trabecular bone in both the tibia and the mandible, the following parameters were measured: bone volume fraction (BV/TV, %), trabecular number (Tb.N, mm^− 1^), trabecular thickness (Tb.Th, mm) and trabecular separation (Tb.Sp, mm). For cortical bone in both sites, BV/TV and cortical thickness (Ct.Th, mm) were assessed.

### Fatty Acid Analysis by High Performance Liquid Chromatography

#### Isolation of Bone Marrow and Mineralized Tissue Fractions

We followed the During protocol to isolate BM and MT fractions [19]. Tibial and mandibular tissues were separated into BM and MT fractions. For the tibia, the medullary canal was initially flushed twice with a water-alcoholic (70% ethanol) solution; this step was omitted for the mandible due to the absence of a medullary cavity. Following fragmentation of both the tibia and mandible, the BM was harvested through two successive washes with the same solution under constant agitation to ensure maximal recovery. To eliminate any residual BM lipids, the remaining bone fragments underwent two additional saline washes with 0.9% NaCl at 40 °C to eliminate any residual bone marrow lipids. Tissues were then cryomilled at − 196 °C into fine powder to preserve fatty acid integrity. All samples were stored at − 80 °C for subsequent lipid extraction. The tibial BM samples were processed to answer other scientific questions and therefore could not be used in the present study.

#### Total Lipid Extraction

Lipids were extracted from BM and MT fractions according to the procedures described previously [19]. Briefly, BM underwent a double-extraction procedure using chloroform/methanol (2:1, v/v) and a theoretical Folch wash, followed by centrifugation and nitrogen evaporation to maximize lipid yield. For MT, additional demineralization steps were performed, involving sequential EDTA chelation (0.5 mol/L) and acid washing (CHCl₃/MeOH/HCl) to release matrix-bound lipids, followed by repeated organic phase recovery and nitrogen evaporation. Final lipid extracts were recovered in small volume (< 400 µL) of chloroform/methanol (2:1; v/v) containing 1% butylated hydroxytoluene (BHT) and kept at − 20 

#### Fatty Acid Naphtacyl Derivatives Preparation

A 19:0 internal standard was added prior to processing to normalize for potential loss during the procedure and allow for quantification. Lipid extracts were subjected to a saponification (50 mg/mL KOH in 95% ethanolic solution, 102 °C, 60 min), then acidified with hydrochloric acid to reach pH 2. Freed FAs were extracted three times with hexane and converted into naphthacyl derivatives by using the derivatization procedure of Rioux et al. [20].

#### High Performance Liquid Chromatography Analysis

FA derivatives were injected into the HPLC system e2695 (Waters Corporation, Milford, USA) equipped with a 2998 photodiode array detector, a reverse-phase YMC PRO C18 column (3 μm, 4.6 × 150 mm, 120 Å; YMC, Kyoto, Japan). FA derivatives were eluted using four solvent systems: (A) methanol/water (64:12, vol/vol), (B) methanol/water (65:7, vol/vol), (C) acetonitrile (100%), and (D) dichloromethane (100%). The elution followed three steps: first, an elution with A/C (76:24, vol/vol) for 10 min, then a linear gradient to replace that solvent by B/D (72:28, vol/vol) for 35 min, and finally an elution with this solvent for 5 min. The flow rate was constant at 1.2 mL/min for the entire run. FA derivatives were detected at 246 nm. For quantification, a nonadecanoic acid (19:0) naphthacyl ester was used to make external calibration curves. Each FA analysis was performed in duplicate in order to reduce the variability of the procedure. The data were collected and processed using Empower 3 (v7.20.00.00; Waters Corporation, Milford, United States) and subsequently reorganized in Microsoft Excel 2021 (Microsoft Corporation, Redmond, United States) before statistical analysis.

Following chromatogram integration, the nineteen main individual FA proportions and concentrations (nmol/g) were determined (see Supplementary Materials, Table S1, for the full list), allowing calculation of SFAs, MUFAs, PUFAs, the *n* − 6/*n* − 3 ratio, and the Δ9 desaturase indices (16:1/16:0, 18:1/18:0).

### Statistical Analysis

Missing data were handled by multiple imputation by chained equations (MICE) using a random forest model; results were consistent with complete-case analyses. Normality was assessed using the Shapiro–Wilk test and by visual inspection of histograms and Q–Q plots. Homogeneity of variances was assessed with an F-test for pairwise comparisons. Data are summarized as mean ± SD for normally distributed variables and as median and interquartile range for non−normally distributed variables. Body weights were compared by repeated-measures two-way ANOVA with Geisser–Greenhouse correction and Šidák’s multiple comparisons. Morphometric and lipid parameters were analyzed with pairwise comparisons independently in each compartment (BM and MT). Those comparisons were conducted with Student’s t-test for normal data and Wilcoxon–Mann–Whitney test for non-normal data. Regarding lipid parameters, fold changes were computed (OVX/SHAM) in each site and compartment to bring variables to a common scale.

To control for multiple testing, p-values were adjusted with the false discovery rate (FDR) using the Benjamini–Krieger–Yekutieli procedure. All analyses were conducted in GraphPad Prism (v10.4.2; GraphPad Software, San Diego, CA, USA). A significance threshold was set at *p* < 0.05.

## Results

Due to non-normal distributions, data are reported as median [Q1–Q3] for all variables.

### Animal Monitoring

No animal was excluded from the experiment.The weight monitoring confirms the metabolic impact of surgery. Statistical analysis revealed a significant effect of hormonal status (*p* = 0.01) and a significant Time × Status interaction (*p* < 0.05), reflecting accelerated weight gain in ovariectomized animals (see Supplementary Materials, Table S2 and Fig. S3).

### Bone Microarchitecture

The microarchitecture was assessed in both trabecular and cortical bone areas for the mandible and tibia (see Table 1). In the tibia, BV/TV and Tb.N were significantly reduced in the OVX group compared to SHAM (− 28% and − 25% respectively, *p* = 0.0003), while Tb.Sp was increased (+ 30%, *p* = 0.0003). In contrast, no significant changes were observed in any trabecular and cortical parameters in the mandible following ovariectomy.

**Table 1:** Microarchitecture of tibial and mandibular cortical and trabecular bone in SHAM and OVX rats

	SHAM	OVX	*p*-value
Tibial Cortical Bone			
Ct.Th (mm)	0.45 [0.42–0.46]	0.42 [0.41–0.44]	0.07
Tibial trabecular bone			
BV/TV (%)	42.9 [41.6–43.9]	30.9 [29.7–33.4]	**0.0003**
Tb.Th (mm)	0.11 [0.10–0.11]	0.10 [0.10–0.11]	0.26
Tb.N (mm^− 1^)	3.97 [3.90–4.01]	2.99 [2.76–3.08]	**0.0003**
Tb.Sp (mm)	0.23 [0.21–0.23]	0.30 [0.27–0.31]	**0.0003**
Mandibular cortical bone			
Ct.Th (mm)	0.38 [0.33–0.46]	0.39 [0.35–0.43]	0.31
Mandibular trabecular bone			
BV/TV (%)	68.9 [60.6–71.5]	62.5 [58.4–72.8]	0.18
Tb.Th (mm)	0.22 [0.17–0.25]	0.23 [0.19–0.26]	0.29
Tb.N (mm^−1^)	3.12 [2.73–3.98]	2.78 [2.53–3.54]	0.16
Tb.Sp (mm)	0.22 [0.15–0.29]	0.20 [0.13–0.29]	0.38

Ct.Th: cortical thickness, BV/TV: bone volume per total volume, Tb.Th: trabecular thickness, Tb.N: trabecular number, Tb.Sp: trabecular separation. SHAM: sham-operated rats (*n* = 10). OVX: ovariectomized rats (*n* = 10). Data are presented as median and interquartile range [Q1–Q3]. Comparisons were done with Wilcoxon–Mann–Whitney test and corrected for multiple tests with False Discovery Rate (FDR). Significant valuess are shown in **bold**.

### Total FA Composition

FA composition was examined in the BM and MT of the mandible and in the MT of the tibia, in order to evaluate the OVX effect (Table 2).

**Table 2:** Fatty acid (FA) composition of mandibular bone marrow (BM) and tibial/mandibular mineralized tissue (MT) (SHAM vs. OVX)

	BM Md SHAM	BM Md OVX	MT Tib SHAM	MT Tib OVX	MT Md SHAM	MT Md OVX	BM Md SHAM vs. OVX	MT SHAM Tib vs. Md	MT Tib SHAM vs. OVX	MT Md SHAM vs. OVX	MT OVX Tib vs. Md
Concentration	3565 [3113–5337]	5979 [4811–6750]	3515 [2739–4737]	5928 [5455–6190]	3505 [2138–4328]	4735 [4280–5102]	**0.01**	0.14	**0.01**	**0.01**	**0.03**
SFA	41.2 [40.2–43.4]	37.2 [36.1–38.7]	35.1 [32.8–36.1]	32.6 [31.2–36.0]	39.1 [38.2–41.4]	37.3 [36.4–37.7]	**0.0003**	**0.0006**	0.20	**0.001**	**0.01**
MUFA	27.6 [25.6–29.2]	31.1 [30.5–32.5]	33.7 [32.6–34.6]	37.8 [37.1–39.2]	28.6 [27.2–30.5]	31.3 [29.6–31.9]	**0.003**	**0.0006**	**0.002**	**0.01**	**0.0003**
PUFA	30.3 [28.2–32.9]	30.8 [29.8–32.8]	31.5 [31.0–32.6]	29.3 [26.3–30.3]	31.0 [29.6–33.8]	32.3 [30.7–33.9]	0.23	0.28	**0.01**	0.24	**0.002**
14:0	1.3 [1.2–1.5]	1.7 [1.5–1.8]	1.8 [1.6–2.1]	2.0 [1.9–2.2]	1.3 [1.1–1.4]	1.7 [1.6–1.9]	**0.005**	**0.002**	0.12	**0.001**	**0.002**
16:0	26.5 [25.3–27.5]	25.0 [23.7–25.8]	24.7 [24.3–25.2]	25.1 [24.1–26.3]	27.1 [24.9–28.2]	24.3 [23.7–25.2]	**0.01**	**0.04**	0.26	**0.01**	0.10
16:1 n-7	2.4 [2.1–3.0]	4.9 [4.6–5.7]	2.3 [2.1–2.6]	6.5 [5.9–6.8]	2.5 [2.0–2.9]	5.2 [5.0–5.9]	**0.0003**	0.28	**0.001**	**0.0003**	**0.002**
18:0	12.9 [11.7–13.6]	9.6 [8.6–10.6]	7.3 [5.4–8.1]	5.2 [3.3–7.0]	10.7 [9.9–12.0]	9.8 [8.9–10.9]	**0.0003**	**0.0009**	0.06	0.11	**0.0006**
18:1 n-9 + n-7	23.5 [22.0–24.8]	25.0 [23.5–26.4]	27.9 [26.1–28.9]	30.1 [29.4–31.1]	25.1 [23.4–26.4]	24.4 [22.6–25.4]	**0.03**	**0.02**	**0.004**	0.19	**0.0003**
18:2 n-6	19.7 [18.7–21.0]	21.3 [20.0–23.3]	25.8 [23.9–27.1]	24.9 [22.7–25.7]	20.2 [18.8–21.7]	21.5 [20.8–21.9]	**0.03**	**0.0009**	0.20	**0.04**	**0.01**
20:4 n-6	7.4 [7.1–8.4]	6.4 [6.0–7.3]	3.9 [3.3–5.3]	2.1 [1.7–2.3]	7.8 [7.4–9.4]	7.9 [7.0–8.4]	**0.04**	**0.004**	**0.001**	0.23	**0.0003**
18:3 n-3	0.9 [0.8–1.0]	1.3 [1.2–1.5]	1.4 [1.2–1.4]	1.7 [1.4–1.8]	0.8 [0.7–0.9]	1.4 [1.2–1.6]	**0.0003**	**0.001**	**0.01**	**0.0003**	**0.03**
22:6 n-3	1.6 [1.3–2.1]	1.0 [0.9–1.2]	0.1 [0.1–0.2]	0.0 [0.0–0.1]	0.8 [0.6–2.2]	0.7 [0.6–1.0]	**0.001**	**0.0006**	**0.01**	0.19	**0.0003**
Total n-6	27.8 [26.1–29.8]	28.4 [27.6–30.3]	30.3 [29.5–31.0]	27.5 [24.8–28.4]	29.3 [28.1–30.7]	30.0 [28.7–31.2]	0.11	0.16	**0.01**	0.19	**0.004**
Total n-3	2.6 [2.2–3.0]	2.4 [2.3–2.5]	1.5 [1.3–1.5]	1.8 [1.5–1.9]	1.8 [1.6–2.9]	2.1 [1.9–2.6]	0.24	**0.004**	**0.02**	0.11	**0.008**
n-6/n-3	11.48 [10.03–11.92]	12.29 [11.54–12.59]	20.68 [19.79–24.01]	15.64 [15.06–16.50]	16.52 [10.59–17.90]	13.68 [12.28–15.93]	**0.04**	**0.003**	**0.001**	0.13	0.06
16:1/16:0	0.09 [0.08–0.11]	0.21 [0.18–0.23]	0.09 [0.08–0.11]	0.25 [0.24–0.27]	0.10 [0.07–0.11]	0.21 [0.21–0.25]	**0.0003**	0.42	**0.001**	**0.0003**	**0.01**
18:1/18:0	1.80 [1.69–2.16]	2.61 [2.27–3.00]	3.89 [3.08–5.50]	5.73 [4.81–9.61]	2.39 [1.94–2.64]	2.48 [2.13–2.82]	**0.0006**	**0.002**	**0.04**	0.24	**0.0003**

BM: bone marrow; MT: mineralized tissue; Md: mandible; Tib: tibia; SHAM: sham-operated rats; OVX: ovariectomized rats; SFA: saturated fatty acids; MUFA: monounsaturated fatty acids; PUFA: polyunsaturated fatty acids. Individual and grouped fatty acids proportions are % of total fatty acids, mol/mol, ratios are mol/mol and concentrations are nmol/g. Data are presented as median and interquartile range[Q1–Q3]. Pairwise comparisons were performed with Wilcoxon-Mann-Whitney test for non-normal distributions. The sum of the category medians maty deviate from 100% (an inherent proporty of the median). Significant values are in **bold**.Sample sizes were: Md SHAM, *n* = 10; Md OVX, *n* = 10; Tib SHAM, *n* = 6; Tib OVX, *n* = 8.

Across compartments, OVX rats showed higher total FA concentrations, with increases in mandibular BM (*p* = 0.01), tibial MT (*p* = 0.01), and mandibular MT (*p* = 0.01).

#### Bone Site Total Fatty Acids Differences in SHAM Condition

**Fig. 2:**
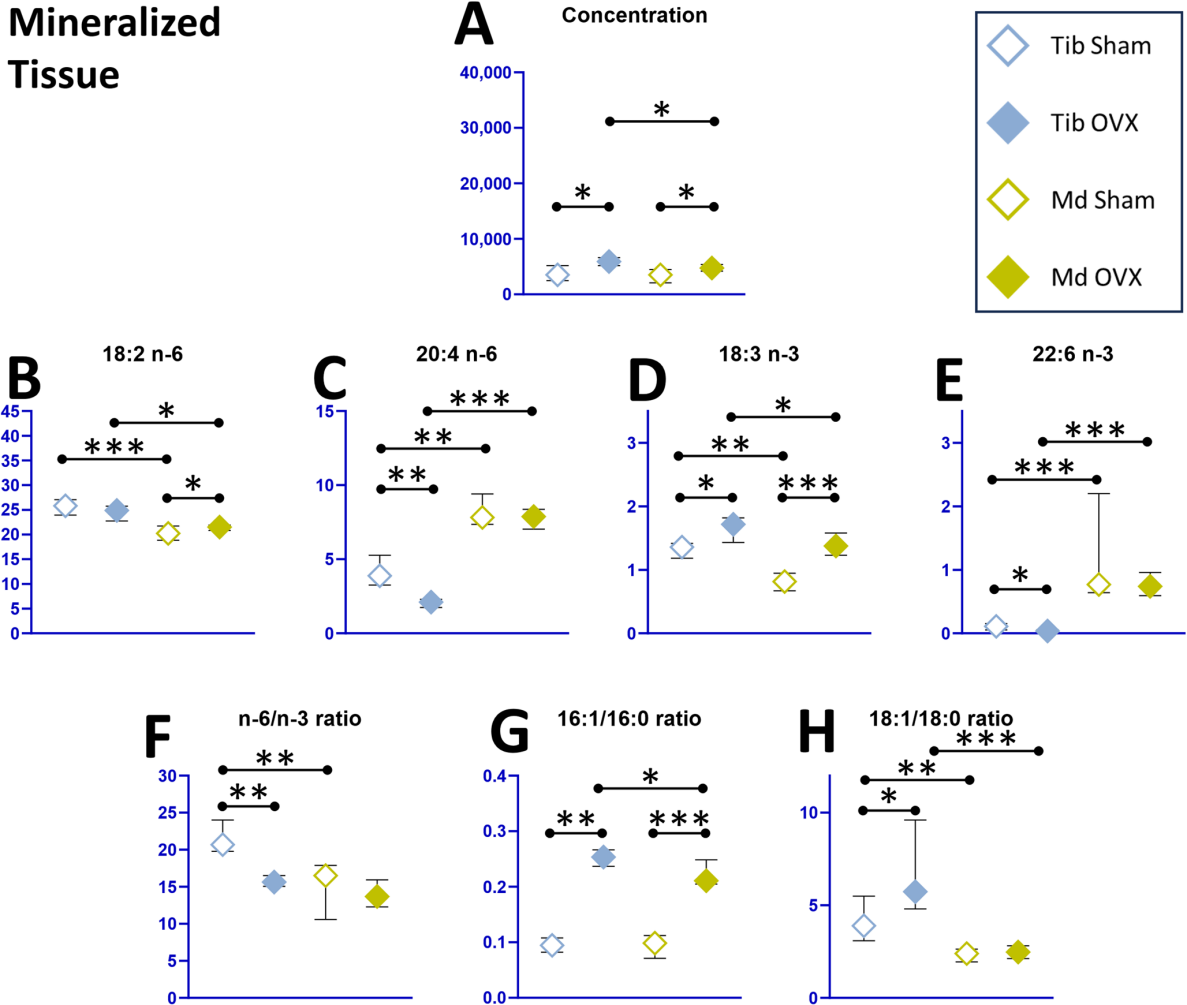
Fatty acid (FA) profile in mandibular vs. tibial mineralized tissue (MT) in SHAM and OVX conditions. Md: mandible; Tib: tibia; SHAM: sham-operated rats; OVX: ovariectomized rats; Individual and grouped fatty acids proportions are % of total fatty acids, mol/mol, ratios are mol/mol and concentrations are nmol/g. Data are presented as median and interquartile range [Q1–Q3]. Pairwise comparisons were performed with Wilcoxon-Mann-Whitney test for non-normal distributions. *p* < 0.05: *; *p* < 0.01: **; *p* < 0.001: ***. Sample sizes were: Md SHAM, *n* = 10; Md OVX, *n* = 10; Tib SHAM, *n* = 6; Tib OVX, *n* = 8

In the SHAM group, total FA concentrations were comparable between mandibular and tibial MT (*p* = 0.14, Fig. 2). Regarding SFAs, mandibular 16:0 and 18:0 proportions were significantly higher than those in the tibia (+ 2 and + 3% points, respectively). Within the MUFA family, 16:1 *n* − 7 levels remained similar between sites, whereas mandibular 18:1 *n* − 9 was lower compared to tibia (− 3 points). PUFAs exhibited distinct site-specific patterns: mandibular linoleic acid (18:2 *n* − 6) and α-linolenic acid (18:3 *n* − 3) were significantly lower compared to tibia (− 22% and − 43%, respectively), while 20:4 *n* − 6 was two-fold higher and docosahexaenoic acid (DHA, 22:6 *n* − 3) was more elevated (0.6–2.2% in the mandible vs. 0.1–0.2% in the tibia). These differences resulted in a significantly lower *n* − 6/*n* − 3 ratio in the mandible (− 20%), driven by the *n* − 3 superiority (+ 0.8 points in 22:6 *n* − 3) without compensation in *n* − 6 levels. Furthermore, while the 16:1/16:0 index was comparable, the 18:1/18:0 desaturation index was significantly lower in the mandible compared to tibia (− 39%). Overall, mandibular MT was proportionally richer in SFAs (+ 4 points) and poorer in MUFAs (− 5 points), while total PUFA levels were maintained across both sites.

#### OVX Effect on Total Fatty Acids Composition

**Fig. 3:**
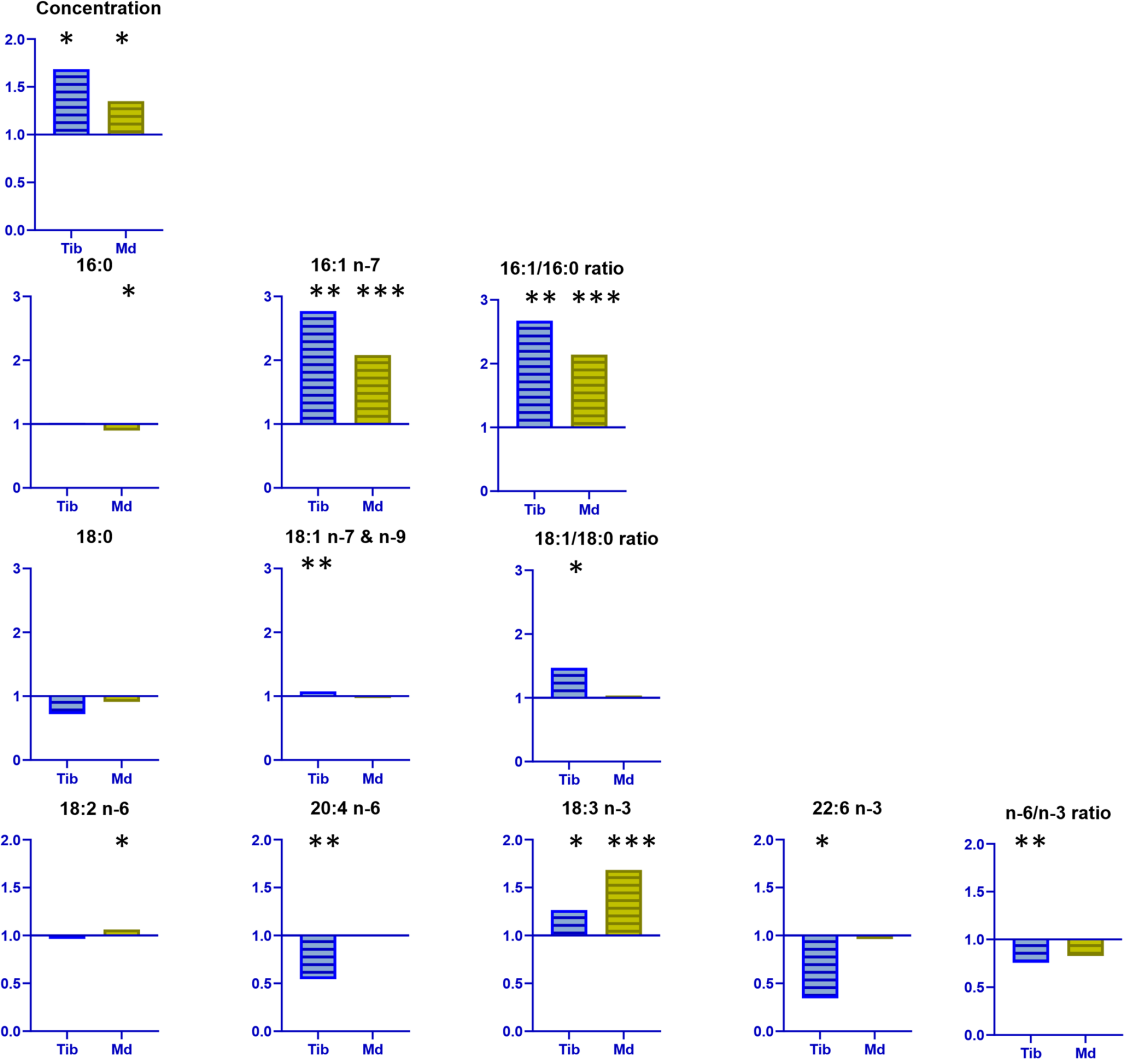
Fold changes in lipid parameters of mineralized tissue (MT) under OVX conditions (relative to SHAM) in mandible and tibia. Md: mandible; Tib: tibia; SHAM: sham-operated rats; OVX: ovariectomized rats; Data are presented as OVX/SHAM fold-changes. SHAM-OVX pairwise comparisons were performed with Wilcoxon-Mann-Whitney test for non-normal distributions. *p* < 0.05: *; *p* < 0.01: **; *p* < 0.001: ***. Sample sizes were: Md SHAM, *n* = 10; Md OVX, *n* = 10; Tib SHAM, *n* = 6; Tib OVX, *n* = 8

Ovariectomy induced an increase in FA concentrations at both skeletal sites (Figs. 2 and 3). In the tibia, the MT from the OVX group showed a large lipid increase compared to SHAM group (+ 69%). A significant increase was also observed in the mandible, although it was quantitatively less pronounced in both MT (+ 35%) and BM (+ 68%) after OVX.

In the tibial MT, the lipid profile showed an enrichment in MUFAs after OVX (+ 4% points), which became the predominant family. This shift occurred at the expense of PUFAs (− 2 points), while SFA proportions remained stable. Individual SFA levels remained stable in the tibia between both groups. In contrast, MUFAs showed increases in both 16:1 *n* − 7 and 18:1 *n* − 9 after OVX (+ 4 and + 2% points, respectively). Within the PUFA family, 18:2 *n* − 6 levels were stable, while 20:4 *n* − 6 significantly decreased (− 46%); furthermore, 18:3 *n* − 3 increased (+ 21%) while 22:6 *n* − 3 was reduced (0.0–0.1% in OVX vs. 0.1–0.2% in SHAM). The *n* − 6/*n* − 3 ratio decreased, driven by both the decline in 20:4 *n* − 6 and the increase in 18:3 *n* − 3 in OVX group compared to SHAM group. Furthermore, in OVX rats the 16:1/16:0 and 18:1/18:0 desaturation indices increased (+ 178% and + 48%, respectively), primarily due to the elevated levels of the MUFAs 16:1 *n* − 7 and 18:1 *n* − 9.

In the mandible, the lipid profile of OVX rats showed a decrease in SFAs in both MT and BM (− 2 and − 4% points, respectively) and an enrichment in MUFAs (+ 3 and + 4 points, respectively), while PUFAs remained stable. Specifically, in the mandibular MT, 16:0 decreased (− 3 points) while 18:0 remained stable, and 16:1 *n* − 7 increased (+ 3 points) while 18:1 *n* − 9 showed no change. In the mandibular BM, both 16:0 and 18:0 decreased (− 2 to − 3 points), whereas 16:1 *n* − 7 and 18:1 *n* − 9 levels increased (+ 2 to + 3 points) in OVX group compared to SHAM group. Regarding PUFAs, 18:2 *n* − 6 increased in both MT and BM (+ 6% and + 8%, respectively). Conversely, 20:4 *n* − 6 decreased in the BM (− 14%) but remained maintained at high levels in the MT after OVX. Similarly, 18:3 *n* − 3 increased (+ 75% and + 44% in MT and BM, respectively), while 22:6 *n* − 3 was reduced in the BM (− 38%) but not in the MT after OVX. In OVX group compared to SHAM group, the *n* − 6/*n* − 3 ratio remained stable in the mandibular MT and slightly increased in the mandibular BM (+ 7%), which is associated with high basal *n* − 3 levels. In the mandibular MT, the 16:1/16:0 ratio increased (+ 110%), while the 18:1/18:0 ratio remained stable, reflecting the consistency of both 18:0 and 18:1 *n* − 9 levels. In the mandibular BM, both 16:1/16:0 and 18:1/18:0 indices increased (+ 133% and + 45%, respectively), driven by the concomitant increase in MUFAs and decrease in SFAs.

## Discussion

### Preserved Mandibular Bone Microarchitecture After Ovariectomy

The success of the ovariectomy was confirmed by visual assessment of the uterine atrophy. It was further confirmed by the significant increase in body weight observed in the OVX group (*p* = 0.01, see Supplementary, Materials Table S2, Figure S3) and by the presence of microarchitectural alterations of the tibia, including a significant decrease in BV/TV and Tb.N as well as an increase in Tb.Sp.

Our results confirm the site-specific resistance of the mandible to estrogen deficiency. While the tibia showed a 28% reduction in BV/TV, the mandible remained structurally unaffected. This aligns with previous reports [17, 21] showing attenuated mandibular changes in mature rats. Discrepancies with studies reporting significant loss [22] may be attributed to rat age and post-OVX duration, as skeletal maturation is critical for observing mandibular stability [23]. This resilience might be supported at the cellular level by compensatory mechanisms like perilacunar osteolysis, which maintains macroscopic architecture despite mineral release [24]. 

### Mandibular Fatty Acids Specificityin the SHAM Group

One of the original features of this work is the parallel characterization of lipids in two mandibular compartments, BM and MT, an approach previously applied to the femur by our group on the same model of ovariectomized rats [14, 19]. We found that in SHAM group, both FA concentrations and profiles were largely similar between the mandibular BM and MT. This observation contrasts with the femur, where BM exhibited a 4- to 7-fold higher total FA concentration than MT [14, 19].

The mandible MT showed lower proportions of 18:2 *n* − 6 and 18:3 *n* − 3, and significantly enriched proportions of 20:4 *n* − 6 and 22:6 *n* − 3 than tibial MT. High dietary intake of 20:4 *n* − 6 has been associated with reduced hip fracture risk in older adults [12]. Dietary enrichment with 20:4 *n* − 6 improved bone mineral content and density in pigs [25]. As a precursor to oxylipins, 20:4 *n* − 6 is metabolized via the lipoxygenase and cytochrome P450 pathways [26, 27]. Its conversion to prostaglandin E2 by cyclooxygenases is essential for mechanotransduction-induced osteogenesis [28, 29], and its inhibition has been shown to impair bone healing in a rabbit model [30]. While 20:4 *n* − 6 is often associated with systemic pro-inflammatory effects in humans [31, 32], its local presence in bone appears to support structural integrity. The high constitutive concentration of 20:4 *n* − 6 in mandibular MT suggests a signaling pool consistent with its high osteogenic potential compared to long bone. This enrichment likely reflects a more active metabolic conversion of its precursor, 18:2 *n* − 6, within this specific site. Furthermore, the higher levels of 22:6 *n* − 3 in the mandible compared to tibia are equally significant. As a primary precursor to specialized pro-resolving mediators (SPMs), including resolvins, protectins, and maresins [27, 33], 22:6 *n* − 3 plays a crucial role in limiting inflammatory damage and promoting tissue repair. By promoting osteoblastic activity while simultaneously inhibiting osteoclastic resorption, a dual effect demonstrated in vitro for both 20:4 *n* − 6 and 22:6 *n* − 3 [34], these essential FA are associated with a local biochemical environment that promotes bone formation and structural strength.

Compared to the tibia, the mandible exhibited a lower *n* − 6/*n* − 3 ratio in SHAM groups, suggesting a lipid environment intrinsically favorable to bone homeostasis. *n* − 3 supplementation promotes SPM production in ovariectomized rats, reinforcing anti-inflammatory pathways within the bone microenvironment [35]. Furthermore, 22:6 *n* − 3 suppresses osteoclast differentiation and resorptive activity in vitro [34]. The mandible’s capacity to maintain a low n **−** 6/n **−** 3 ratio is consistent with favorable remodeling dynamics observed in this model.

In mandibular MT, we observed a lower 18:1/18:0 ratio compared to the tibia, consistently lower than the femoral values reported by During [14]. This is consistent with the mandibular enrichment in 20:4 *n* − 6, known to inhibit the Δ9 desaturase SCD-1 activity [36]. Reduced Δ9 desaturase activity in the mandibular MT may impair adipocyte storage within BMAT, and contribute to the specific mandibular signature of reduced BMAT compared with long bones, a trend we recently highlighted in a systematic review [18]. The reduced SCD-1 activity possibly rooted in the mandible’s distinct ectodermic origin [37], favors high energy consumption over lipid storage.

By combining restricted Δ9 desaturase indices with an enrichment in pro-resolving PUFAs, the mandible maintains a biochemical environment inherently less susceptible to adverse remodeling.

### OVX Effect on the Total FA Composition

In the tibia, MT total FA content increased (+ 69%), reaching6,000 nmol/g, whereas the mandible showed a more modest increase (+ 35% to 4,700 nmol/g in MT, + 68% to 6,000 nmol/g in BM). This limited expansion is consistent with the lack of significant BMAT development in the mandibular condyle and alveolar regions compared to the large adipose infiltration observed in long bones in a rat model [17]. Given the strong negative correlation between BMAT and BV/TV in tibia of OVX rats [9], this restricted adipose expansion appears to be a factor in mandibular structural resistance. To date, the only other studies characterizing the MT lipid profile in OVX rats were conducted by During et al. [14] and Delattre et al. [16], who reported a substantial 2- to 3-fold increase in total FA concentration within the femur. Because mandibular FA levels are significantly lower in SHAM group than those in the tibia, the increase in the mandible OVX group may not reach the critical threshold necessary to induce structural alterations. This protection may be linked with a reduction in local lipotoxicity suggested by the lower lipid concentrations observed. In the tibia, BMAds can cover up to 87% of the trabecular surface, whereas they remain relatively isolated in the mandible (5% coverage) [17]. This anatomical distance, reinforced by the mandible’s predominantly cortical structure, limits the paracrine exposure of osteoblasts to deleterious SFAs like 16:0 and 18:0, known to inhibit mineralization and induce osteoblast apoptosis in vitro [38, 39].

The increase of 18:3 *n* − 3 observed in the MT of both the tibia and the mandible post-OVX may represent a localized compensatory response. 18:3 *n* − 3 has been shown to moderate the deleterious effects of estrogen deficiency in vitro and in vivo [27], as well as on rat mandibular bone [40]. While the mandible exhibited lower 18:3 *n* − 3 levels than the tibia, its total *n* − 3 PUFA content remained superior. This is consistent with evidence that *n* − 3 PUFAs enhance osteogenesis and bone resilience in rat models, including the alveolar bone where they reduce inflammation and bone loss [41]. Furthermore, the stability of 22:6 *n* − 3 in the mandibular MT contrasts to its depletion in the BM and its overall scarcity in the tibia. As a primary precursor for SPMs, the maintenance of 22:6 *n* − 3 levels in the mandibular mineralized tissue likely preserves a robust anti-inflammatory balance [27].

The *n* − 6/*n* − 3 ratio, while frequently employed as a marker of inflammatory balance, presents significant interpretative challenges in bone biology due to its association with both protective and deleterious skeletal effects [42]. In the tibial MT, this ratio decreased following OVX primarily because of a significant reduction in 20:4 *n* − 6, whereas the mandible maintained stable levels of both the ratio and this specific FA. This stability is particularly relevant given that 20:4 *n* − 6 concentrations correlate positively with Ct.Th and negatively with porosity in the OVX rat femur [16], consistent with findings that its dietary enrichment has been shown to enhance bone quality in piglet [25] and reduce fracture risk in human [12]. The preservation of 20:4 *n* − 6 in the mandible suggests a resilient signaling pool of oxylipin precursors, facilitating superior remodeling dynamics compared to the tibia and femur, where these levels are typically depleted post-OVX [11, 14, 35]. Beyond the ratio itself, the stability of total *n* − 3 content in the mandible supports an environment geared toward an anti-inflammatory balance. While some evidence suggests that *n* − 6 PUFAs may promote bone resorption, *n* − 3 supplementation has been shown to reduce PGE_2_ production and inhibit osteoclastogenesis [43]. This is consistent with findings in transgenic FAT-1 mice, where endogenous conversion of *n* − 6 to *n* − 3 prevented OVX bone loss and reduced BMAT expansion by suppressing osteoclast differentiation [44]. In the mandible, the high and stable *n* − 3 content likely maintains a constitutive anti-inflammatory state, whereas the ratio decrease observed in the tibia likely reflects a late-stage compensatory adaptation to estrogen deficiency rather than a primary protective mechanism. By maintaining high concentrations of precursors like 20:4 *n* − 6 and 22:6 *n* − 3, the mandible retains a superior capacity for inflammatory modulation and bone repair through oxylipin-mediated signaling.

In osteoporotic conditions, previous studies have consistently identified a shift from SFAs to MUFAs. Miranda et al. [11] reported reduced SFAs and elevated MUFAs in the BM fluid of women with a fracture history, a pattern reinforced by During et al. [14] with elevated MUFAs in the MT of OVX rats. Our findings confirm that the tibia undergoes this characteristic osteoporotic transition, showing higher 16:1 *n* − 7 and 18:1 *n* − 9 levels alongside significantly increased Δ9 desaturase indices. In contrast, mandibular MT maintains a lipid environment naturally enriched in SFAs that persists following OVX. While 16:1 *n* − 7 increased in the mandible, 18:1 *n* − 9 remained unchanged, resulting in a stable 18:1/18:0 index. This distinction is significant, as elevated 18:1/18:0 ratios are linked to bone impairment in both human and rodent models [14–16]. Specifically, Delattre et al. [16] established the 18:1/18:0 ratio as a detrimental marker of bone health, correlating negatively with cortical thickness and positively with cortical porosity, while identifying 20:4 *n* − 6 as a beneficial marker. Interestingly, 20:4 *n* − 6 levels displayed an inverse relationship, as their depletion following OVX was directly associated with cortical deterioration. Given that this PUFA is a known inhibitor of the Δ9 desaturase [45], our findings reinforce the hypothesis of a potential association between the preservation of 20:4 *n* − 6 and the restricted enzymatic desaturation observed in the mandible, which may collectively contribute to its reduced OVX-induced bone loss.

Ultimately, while ovariectomy induced lipid remodeling in the long bones, it triggered only modest changes in the mandible. The preservation of high 20:4 *n* − 6 and 22:6 *n* − 3, and lower Δ9 desaturase indices, characterize the mandibular response. Pathways involving the *de novo* synthesis of 18:1 from 18:0 and the catabolism of 20:4 *n* − 6 have been proposed as central markers of cortical bone loss [16]. In this context, the high 20:4 *n* − 6 content maintained in mandibular MT may reflect a reduced conversion to downstream pro-inflammatory oxylipins, despite the abundance of available substrate. Such attenuated remodeling and the maintenance of a biochemically lean environment likely characterize the mandible’s relative protection and structural resilience in the face of systemic estrogen deficiency.

### Necessity of Follow-up Studies

This study offers novel insights into the mandibular lipid signature, yet some methodological constraints and the exploratory nature of the design must be acknowledged. The observational design of this study, conducted at a single endpoint, focuses on establishing associations rather than defining direct causative mechanisms. These findings provide a foundational framework for future longitudinal studies to track the temporal progression of such metabolic shifts. Subsequent mechanistic research, incorporating direct assessments of lipid metabolism and enzymatic activity, will be essential to elucidate whether these lipid alterations actively contribute to bone pathology or function as descriptive biomarkers of systemic estrogen deficiency.

Animals were sourced from a separate study in accordance with the 3Rs principles (Reduction) by minimizing the number of animals used. Consequently, no a priori power analysis was performed to determine a formal sample size. Furthermore, as the experimental design was primarily powered to detect treatment effects rather than differences between bone compartments, no formal statistical comparisons between BM and MT were conducted. These specific comparisons should therefore be regarded as exploratory and hypothesis-generating. Additional limitations include the use of a single disease model (estrogen deficiency), a single time point, and the lack of functional endpoints.

A primary limitation of this study is the absence of tibial BM analysis, which resulted in an asymmetric experimental design. This was necessitated by the unavailability of the tibial BM, used in a separate investigation. The lipid profiles observed in the tibial MT align with our previous findings regarding femoral MT in OVX rats [14]. In both long bones, MT demonstrated a characteristic increase in FA concentrations and MUFA enrichment, coupled with elevated Δ9 desaturase indices and diminished levels of 20:4 *n* − 6 and 22:6 *n* − 3. These parallel findings support the hypothesis that the tibial BM response mirrors the femoral BM profile, which was previously characterized by marked FA accumulation, increased Δ9 desaturase indices, and a decline in 20:4 *n* − 6 and 22:6 *n* − 3, as well.

To estimate Δ9 desaturase (SCD-1) activity, we used product-to-precursor ratios (16:1/16:0 and 18:1/18:0), which are standard proxies in lipid research [16, 45]. The presence of SCD-1 protein in the rat BM has been shown [46], and its enzymatic activity is linked to metabolic pathways including de novo lipogenesis and β-oxidation in adipocytes [45, 47]. Given that the 16:1/16:0 ratio was associated with fracture risk in men [15], as well as a higher 18:1/18:0 ratio was observed in bone marrow fluid from post-menopausal women with hip fractures, compared to those without fracture [11]. These indices provide valuable insight into the lipid profiles of resilience across different skeletal sites following ovariectomy.The mandible’s relative resistance to this expansion of bone marrow adipose tissue observed in long bones during estrogen deficiency makes it a unique biological model for bone resilience. Clinically, these findings suggest that the mandibular lipidome could serve as a metabolic reference for developing targeted therapies. By understanding how this specific site preserves bone quality and microarchitectural integrity even under systemic stress, we may identify novel pharmacological targets to replicate this protective effect in more vulnerable skeletal sites, such as the femoral neck or vertebrae.

Regarding generalizability, the site-specific mandibular lipid signature and attenuated bone impairment in the rat align with clinical observations that craniofacial bones can be less susceptible to estrogen-deficiency changes, suggesting plausible relevance to human craniofacial biology. However, species differences in remodeling dynamics constrain direct extrapolation to humans. Future work should therefore consider larger cohorts, longitudinal designs, high-resolution regional analyses within the mandible, and interventional approaches (dietary or pharmacological), in order to establish causal links between FA pathways and skeletal remodeling.

## Conclusions

In this SHAM-controlled OVX model, lipid remodeling was pronounced in the tibia but attenuated and qualitatively distinct in the mandible. Quantitatively, the total fatty acids were higher in mineralized tissue in OVX groups, with a stronger increase in the tibia (+ 69%) than in the mandible (+ 35% in MT), underscoring the mandible’s reduced lipid accumulation under estrogen deficiency within this compartment. Qualitatively, in the OVX group the tibia MT displayed the typical osteopenic signature, with reduced 20:4 *n* − 6 level and elevated Δ9 desaturase indices, whereas the mandible MT retained a high and stable 20:4 *n* − 6 level, while 18:3 *n* − 3 was higher and 22:6 *n* − 3 remained stable, as well as a stable 18:1/18:0 ratio despite an OVX-associated rise in 16:1/16:0. Together, these findings demonstrate site-specific lipid responses to estrogen deficiency and highlight three protective mandibular traits, attenuated adiposity, stable Δ9 desaturation index (18:1/18:0), and unchanged 20:4 *n* − 6 level, while reinforcing Δ9 desaturation and arachidonic acid metabolism as candidate pathways for further mechanistic investigation. Given the single-model, single-time-point design and the absence of oxylipin and functional endpoints, follow-up studies incorporating longitudinal sampling, regional mandibular mapping, oxylipin and enzyme assays, and interventional approaches are required to establish causal links between lipid pathways and skeletal remodeling.

## Supplementary Information

Below is the link to the electronic supplementary material.

Supplementary Material 1

Supplementary Material 2

Supplementary Material 3

Supplementary Material 4

Supplementary Material 5

Supplementary Material 6

Supplementary Material 7

Supplementary Material 8

## Data Availability

The data that support the findings of this study are available from the corresponding author, Maxime Bedez, upon reasonable request.

## Abbreviations

BHT: Butylated hydroxytoluene
BM: Bone marrow
BMAd: Bone marrow adipocyte
BMAT: Bone marrow adipose tissue
BMD: Bone mineral density
BV/TV: Bone volume per total volume
Ct.Th: Cortical thickness
DHA: Docosahexaenoic acid
HPLC: High performance liquid chromatography
MT: Mineralized tissue
MUFA: Monounsaturated fatty acid
OVX: Ovariectomy/Ovariectomized
PGE_2_: Prostaglandin E_2_
PUFA: Polyunsaturated fatty acid
ROI: Region of interest
SFA: Saturated fatty acid
SPM: Specialized pro-resolving mediators
Tb.N: Trabecular number
Tb.Sp: Trabecular separation
Tb.Th: Trabecular thickness
