# Supplementary material for: Preserved Mandibular Bone Microarchitecture Following Ovariectomy-induced Osteoporosis is Associated with a Specific Fatty Acid Composition in the Rat
Source: Calcif Tissue Int. 2026 May 7;117(1):77. doi: 10.1007/s00223-026-01543-5 (PMC13152906; doi:10.1007/s00223-026-01543-5)
Supplement: Supplementary file 1 — Supplementary Material 1 [file 223_2026_1543_MOESM1_ESM.docx]

# OSTEOMAN

# Supplementary Materials

## Fig. S1: Regions of Interest in Tibia and Mandible


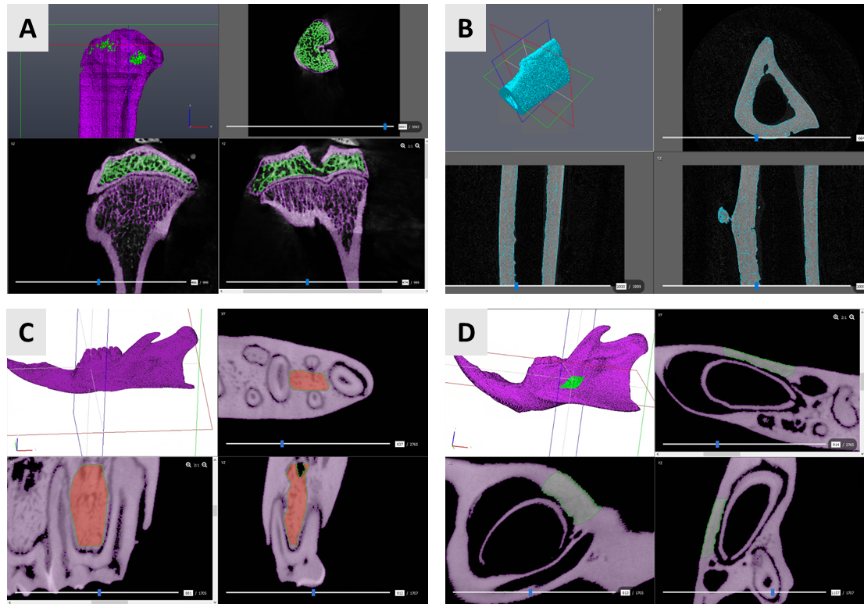


Figure S1: Regions of interest (ROIs). (A) Trabecular tibia: secondary ossification center of the proximal epiphysis. (B) Cortical tibia: mid-diaphysis. (C) Trabecular mandible: interradiculair septum of the first molar. (D) Cortical mandible: buccal cortical plate, distal to the roots of the third molar.

## Fig. S2: Thermal exchanger for Cryo-µCT


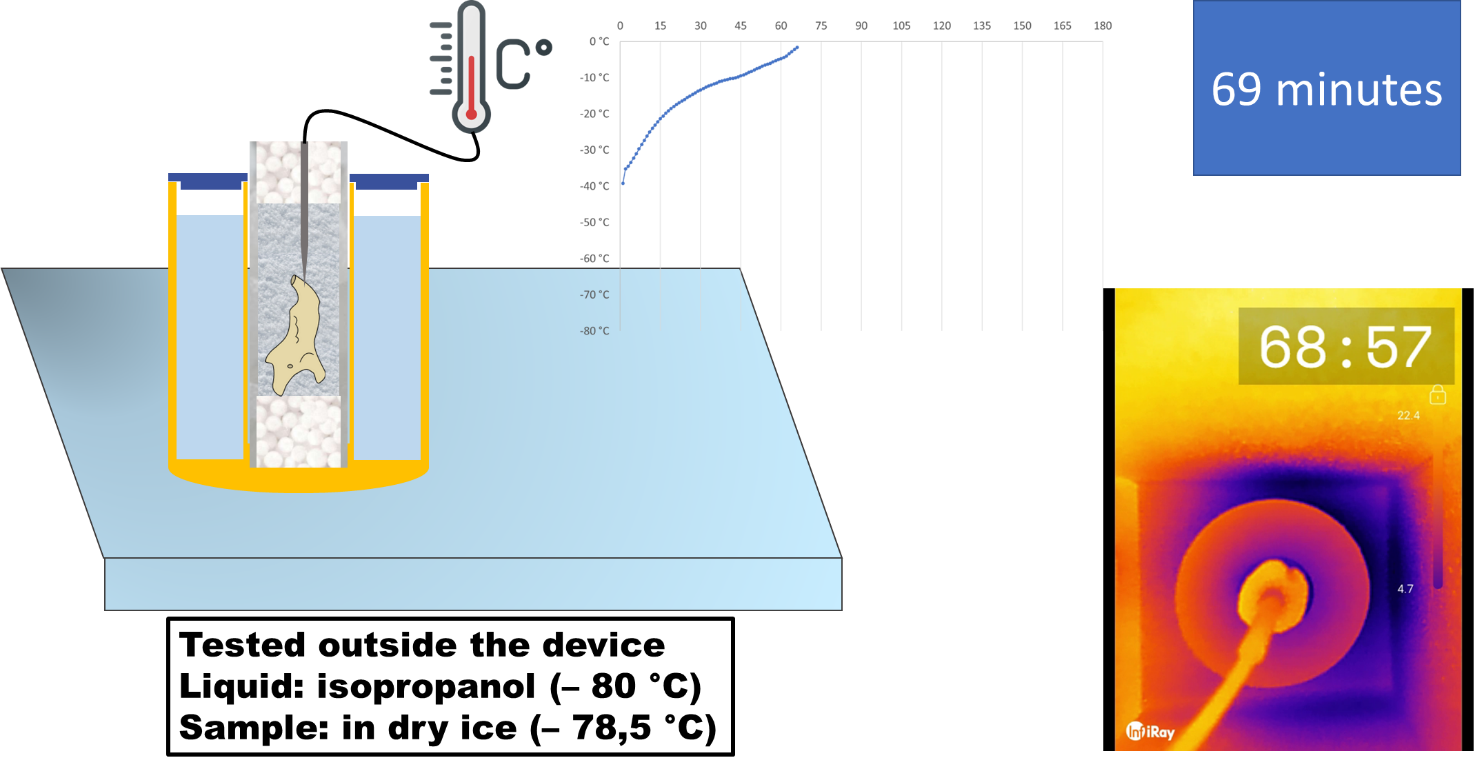


Figure S2: Thermal exchanger model (last prototype) showing a theorical 69 minutes durations under 0 °C, made to fit the maximum of cold refrigerant (isopropanol) in the Skyscan 1172 micro-CT device. The .stl file for impression is available on reasonnable request to the corresponding author.

## Table S1: Fatty acids Detected

Table S1: Fatty acids detected and quantified with our method of HPLC chromatography. 19:0 is a non-naturally occurring fatty acid used as a standard. 18:1 n−9 and 18:1 n−7 are quantified as a single peak. n−6/n−3 ratio is calculated with (18:2 n−6 + 20:2 n−6 + 20:3 n−6 + 20:4 n−6) / (18:3 n−3 + 20:3 n−3 + 20:5 n−3 + 22:6 n‑3).

|  | Abreviated name | Trivial name | IUPAC name |
| --- | --- | --- | --- |
| Saturated | 14:0 | Myristic acid | tetradecanoic acid |
| fatty acids | 15:0 | Pentadecylic acid | pentadecanoic acid |
|  | 16:0 | Palmitic acid | hexadecanoic acid |
|  | 17:0 | Margaric / heptadecylic acid | heptadecanoic acid |
|  | 18:0 | Stearic acid | octadecanoic acid |
|  | 19:0 | Nonadecylic acid | nonadecanoic acid |
|  | 20:0 | Arachidic acid | eicosanoic acid |
| Monounsaturated | 16:1 n−7 | Palmitoleic acid | cis-9-hexadecenoic acid |
| fatty acids | 18:1 n−9 | Oleic acid | cis-9-octadecenoic acid |
|  | 18:1 n−7 | Vaccenic acid | trans-11-octadecenoic acid |
|  | 18:1tr n−9 | Elaidic / trans-oleic acid | trans-9-octadecenoic acid |
|  | 20:1 n−9 | Eicosenoic acid | cis-11-eicosenoic acid |
|  | 22:1 n−9 | Erucic acid | cis-13-docosenoic acid |
|  | 24:1 n−9 | Nervonic acid | cis-15-tetracosenoic acid |
| Polyunsaturated | 18:2 n−6 | Linoleic acid | cis-9,12-octadecadienoic acid |
| fatty acids | 18:3 n−3 | α-linolenic acid | cis-9,12,15-octadecatrienoic acid |
|  | 20:2 n−6 | Docosadienoic acid | cis-13,16-docosadienoic acid |
|  | 20:3 n−3 | Eicosatrienoic acid | cis-11,14,17-eicosatrienoic acid |
|  | 20:3 n−6 | DGLA acid | cis-8,11,14-eicosatrienoic acid |
|  | 20:4 n−6 | Arachidonic acid / AA / ARA | cis-5,8,11,14-eicosatetraenoic acid |
|  | 22:6 n−3 | Docosahexaenoic acid / DHA | cis-4,7,10,13,16,19-docosahexaenoic acid |

## Table S2 & Fig. S3: Animal Weights

Table S2: Mean animal weights (g), per week and per group

|  | **OVX** | **SD** | **SHAM** | **SD** |
| --- | --- | --- | --- | --- |
| W0 | 337 | 27 | 330 | 39 |
| W1 | 370 | 30 | 331 | 41 |
| W2 | 388 | 28 | 335 | 34 |
| W3 | 396 | 30 | 351 | 38 |
| W4 | 407 | 31 | 355 | 41 |
| W5 | 414 | 32 | 361 | 49 |
| W6 | 421 | 33 | 361 | 48 |
| W7 | 426 | 35 | 366 | 47 |
| W8 | 429 | 35 | 368 | 47 |
| W9 | 433 | 37 | 372 | 46 |
| W10 | 436 | 36 | 380 | 49 |
| W11 | 439 | 37 | 384 | 51 |
| W12 | 437 | 36 | 376 | 55 |
| W13 | 444 | 36 | 386 | 53 |
| W14 | 448 | 38 | 386 | 54 |
| W15 | 453 | 37 | 392 | 57 |
| W16 | 457 | 37 | 398 | 59 |
| W17 | 458 | 38 | 398 | 63 |
| W18 | 458 | 38 | 403 | 65 |
| W19 | 464 | 37 | 409 | 67 |
| W20 | 468 | 37 | 412 | 68 |
| W21 | 469 | 38 | 415 | 70 |
| W22 | 471 | 40 | 418 | 74 |

*SHAM: sham-operated rats (n = 10). OVX: ovariectomized rats (n = 10). SD: standard deviation. W: week. All values are indicated in grams. Rats were euthanized on week 22.*

The two‑way repeated‑measures ANOVA gave p-values of 0.013 for the SHAM/OVX, < 0.001 for the time and < 0.001 for the Time x SHAM/OVX.


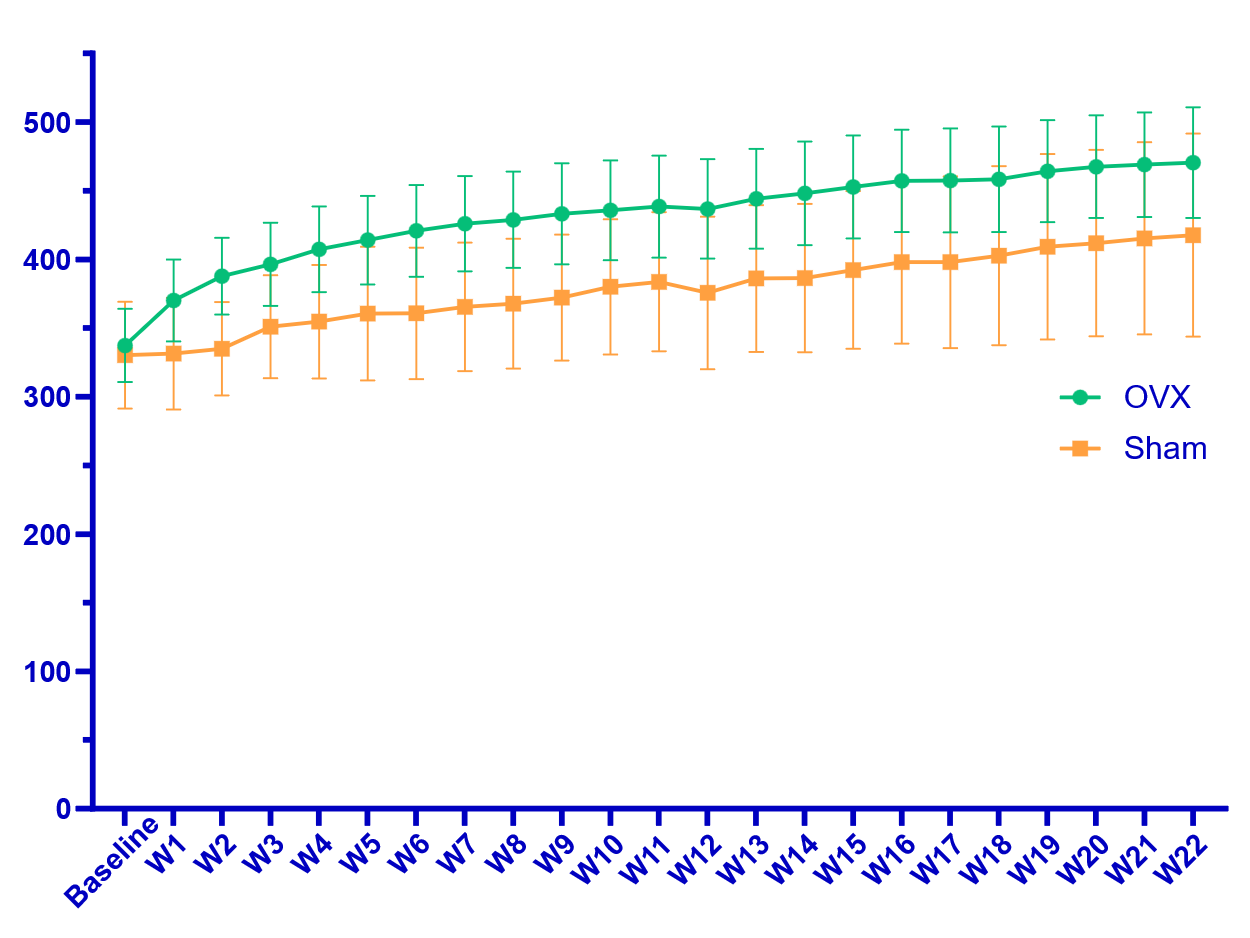


Figure S3: Animal weight monitoring during the OSTEOMAN study according to surgical status. SHAM: sham-operated rats (n = 10). OVX: ovariectomized rats (n = 10). Data are presented as mean ± standard deviation (SD). W: weeks since the start of the study. All values are expressed in grams. Euthanasia was performed at week 22.
