# Supplementary material for: Preserved Mandibular Bone Microarchitecture Following Ovariectomy-induced Osteoporosis is Associated with a Specific Fatty Acid Composition in the Rat
Source: Calcif Tissue Int. 2026 May 7;117(1):77. doi: 10.1007/s00223-026-01543-5 (PMC13152906; doi:10.1007/s00223-026-01543-5)
Supplement: Supplementary file 2 — Supplementary Material 2 [file 223_2026_1543_MOESM2_ESM.docx]

# Review 2026.04.05

***We have meticulously addressed all reviewer comments by clarifying our methodological rationale, refining our interpretations to better reflect the observational nature of the data, and correcting a minor concentration discrepancy. We believe these revisions have significantly enhanced the clarity and rigor of our work, and we look forward to your further evaluation of this manuscript for publication in Calcified Tissue International.***

## Reviewer #1

### Introduction

The primary objective of this study was to examine the fatty acid profile of the mandibular bone to ascertain its biochemical signature in ovariectomized rats, as compared to Sham-control rats, and as compared to the tibial bone site. The authors concluded that the fatty acid composition is site-specific. Furthermore, regarding this composition, the ovariectomy induced only slight changes in the mandible, in contrast to the tibia.

This is an interesting explanatory work that offered new insights into the mandibular lipid signature in control as well as in ovariectomized rats.  This work is well designed and well discussed. But several issues minor the interest of this study and need to be taken into consideration.

### • Reviewer #1, Comment 1

The section entitled "Background" should be renamed "Introduction."

We appreciate this structural clarification and have updated the section heading accordingly.

### • Reviewer #1, Comment 2

In order to determine the efficacy of the ovariectomy, it is necessary to provide the hormonal biochemical status of the animals in both groups, with particular attention to the beta-estradiol status.

We appreciate this insightful suggestion. It is indeed central to our study design that the ovariectomy (OVX) is rigorously validated.

Since 1994, the FDA has recommended the ovariectomized rat as a primary preclinical species for evaluating postmenopausal osteoporosis [1]. The suitability of this model for studying mandibular bone loss was further validated by Johnston and Ward (2015) [2].

In this study, we used the Sprague-Dawley rat as a conventional animal model. The OVX procedure was performed by the provider (Charles River) prior to transfer to our facility. We confirmed the efficacy of the procedure through visual assessment of uterine atrophy during tissue harvest, as well as by monitoring body weight gain and significant changes in tibial bone microarchitecture (decreased BV/TV and Tb.N, increased Tb.Sp).

To ensure clarity regarding the model's validity, we have added these details to the Discussion section.

| Line (old) | Original | Modification |
| --- | --- | --- |
| 283 | - | The success of the ovariectomy was confirmed by visual assessment of the uterine atrophy. It was further confirmed by the significant increase in body weight observed in the OVX group (p = 0.01, see Supplementary Materials Table S2, Figure S3) and by the presence of microarchitectural alterations of the tibia, including a significant decrease in BV/TV and Tb.N as well as an increase in Tb.Sp. |

### • Reviewer #1, Comment 3

Why FA composition was not investigated in the bone marrow of tibia? This will also allow for comparisons between the two groups at this site.

***Note: this answer is duplicated for Reviewer #1, Comment 3 and Reviewer #2, Comment 2.***

The reviewer raises a valid point regarding transparency; we agree that clarifying the sampling rationale improves the manuscript's context.

The bone marrow of tibia was not available. This research was indeed developed in coordination with an ancillary study, which provided the opportunity to specifically examine the mandibular lipid profile in OVX condition compared to SHAM condition. We chose to focus on the mandible not only due to this availability but also because of its unique anatomical features; specifically, the absence of a medullary canal makes its biological organization distinct from long bones like the tibia.

Regarding the comparison with the tibia, we utilized the available mineralized tissue (MT) fraction as a secondary objective to build upon our previous laboratory data on long bone [3]. While we acknowledge that this comparison is limited to the MT compartment, we believe these findings offer a valuable complementary perspective.

In accordance with the reviewer's suggestion, we have revised the Objectives and Discussion sections to more explicitly describe the sampling rationale and the resulting focus of our analysis.

| Line (old) | Original | Modification |
| --- | --- | --- |
| 51-55 | In female Sprague-Dawley rats, we investigated whether estrogen deficiency induced by ovariectomy, compared to sham-operated (SHAM) controls and tibial sites, affects the mandibular fatty acid profile, including individual proportions and ratios in both MT and BM. A secondary question addressed the site effect, namely whether mandibular and tibial lipid profiles differ under both SHAM and OVX conditions. | In female Sprague-Dawley rats, we investigated whether estrogen deficiency induced by ovariectomy, compared to sham-operated (SHAM) controls ~~and tibial sites~~, affects the mandibular fatty acid profile, including individual proportions and ratios in both MT and BM. A secondary question addressed the site effect, namely whether mandibular and tibial lipid profiles differ under both SHAM and OVX conditions in MT. |
| 130-131 | Tibial BM samples were reserved for a separate investigation and were not analyzed in the present study. | The tibial BM samples were processed to answer other scientific questions and therefore could not be used in the present study. |
| - | - | A primary limitation of this study is the absence of tibial BM analysis, which resulted in an asymmetric experimental design. This was necessitated by the unavailability of the tibial BM, used in a separate investigation.  The lipid profiles observed in the tibial MT align with our previous findings regarding femoral MT in OVX rats [3]. In both long bones, MT demonstrated a characteristic increase in FA concentrations and MUFA enrichment, coupled with elevated Δ9 desaturase indices and diminished levels of 20:4 n−6 and 22:6 n−3. These parallel findings support the hypothesis that the tibial BM response mirrors the femoral BM profile, which was previously characterized by marked FA accumulation, increased Δ9 desaturase indices, and a decline in 20:4 n−6 and 22:6 n−3, as well.  While the mandibular BM followed a comparable metabolic trajectory, characterized by significant FA accumulation and similar shifts in desaturase activity, less pronounced than those observed in the femur [3]. Ultimately, although the mandible displays directional shifts similar to the femur and tibia, its distinct baseline and final lipid levels underscore a site-specific physiological response that distinguishes the mandible from the long bones. |

### • Reviewer #1, Comment 4

In order to enhance the interest of this study, it is essential to discuss the clinical relevance of its findings, with a particular emphasis on protective mandibular traits.

To further enhance the clinical relevance of our findings, as thoughtfully suggested, we have added a dedicated paragraph in the Discussion section, addressing the mandible’s clinical relevance as a model for bone resilience. We now highlight its preserved bone quality and unique lipidome as a "metabolic reference" for developing therapies for more vulnerable sites, such as the femoral neck or vertebrae.

| Line (old) | Original | Modification |
| --- | --- | --- |
| - | - | The mandible’s relative resistance to this expansion of bone marrow adipose tissue observed in long bones during estrogen deficiency makes it a unique biological model for bone resilience. Clinically, these findings suggest that the mandibular lipidome could serve as a metabolic reference for developing targeted therapies. By understanding how this specific site preserves bone quality and microarchitectural integrity even under systemic stress, we may identify novel pharmacological targets to replicate this protective effect in more vulnerable skeletal sites, such as the femoral neck or vertebrae. |

### Conclusion

In conclusion, although interesting in some aspects, several issues must be considered before a potential publication in CTIN.

***We trust that these revisions, specifically the strengthened validation of the OVX model and the expanded discussion on clinical relevance, successfully address the reviewer’s valuable suggestions and enhance the overall impact of the study.***

## Reviewer #2

### Introduction

This manuscript addresses a relevant and interesting question. The combined analysis of bone microarchitecture and fatty acid profiles is conceptually appropriate, and the observation that the mandible remains structurally preserved while the tibia undergoes clear osteoporotic changes is consistent with prior evidence.

However, a few important limitations weaken the strength of the conclusions.

### • Reviewer #2, Comment 1

The main concern is the interpretation of the data. The study is observational and conducted at a single endpoint, yet the discussion suggests that lipid composition may contribute mechanistically to mandibular resistance. While this is presented cautiously, the experimental design does not allow causal inference. The analyses are limited to compositional lipid measurements, without direct assessment of lipid metabolism, enzymatic activity, or temporal dynamics. Therefore, the data support association rather than mechanism.

We thank the reviewer for this constructive feedback. We fully acknowledge that the cross-sectional and observational nature of our study, conducted at a single endpoint, precludes any direct causal inference. To address this, we have carefully refined the phrasing throughout the Results and Discussion sections to ensure that our findings are framed as associations rather than mechanistic links. We have also expanded the Discussion to explicitly state the study's limitations. We believe these revisions now accurately reflect the descriptive and foundational nature of our data, providing a more nuanced interpretation of the mandibular lipid profile.

| Line (old) | Original | Modification |
| --- | --- | --- |
| 307-309 | The high constitutive concentration of 20:4 n−6 in mandibular MT suggests a signaling pool that may sustain its high osteogenic potential compared to long bone. | The high constitutive concentration of 20:4 n−6 in mandibular MT suggests a signaling pool consistent with its high osteogenic potential compared to long bone. |
| 313-316 | By promoting osteoblastic activity while simultaneously inhibiting osteoclastic resorption, a dual effect demonstrated in vitro for both 20:4 n−6 and 22:6 n−3 [4], these essential FA contribute to a local biochemical environment that promote bone formation and structural strenght. | By promoting osteoblastic activity while simultaneously inhibiting osteoclastic resorption, a dual effect demonstrated in vitro for both 20:4 n−6 and 22:6 n−3 [4], these essential FA are associated with a local biochemical environment that promotes bone formation and structural strength. |
| 321-322 | The mandible's capacity to maintain a low n−6/n−3 ratio likely preserve favorable remodeling dynamics. | The mandible's capacity to maintain a low n−6/n−3 ratio is consistent with favorable remodeling dynamics observed in this model. |
| 324-325 | This may be locally regulated by the mandibular enrichment in 20:4 n−6, known to inhibit the Δ9 desaturase SCD-1 activity [5]. | This is consistent with the mandibular enrichment in 20:4 n−6, known to inhibit the Δ9 desaturase SCD-1 activity [5]. |
| 346-347 | This protection is likely mediated by a reduction in local lipotoxicity. | This protection may be linked with a reduction in local lipotoxicity suggested by the lower lipid concentrations observed. |
| 397-401 | Given that this PUFA is a known inhibitor of the Δ9 desaturase [45], our findings reinforce the hypothesis of a functional link between the preservation of 20:4 n−6 and the restricted enzymatic desaturation observed in the mandible, which may collectively contribute to its reduced OVX-induced bone loss. | Given that this PUFA is a known inhibitor of the Δ9 desaturase [45], our findings reinforce the hypothesis of a potential association between the preservation of 20:4 n−6 and the restricted enzymatic desaturation observed in the mandible, which may collectively contribute to its reduced OVX-induced bone loss. |
| 408-410 | Such attenuated remodeling and the maintenance of a biochemically lean environment likely underpin the mandible's relative protection and structural resilience in the face of systemic estrogen deficiency. | Such attenuated remodeling and the maintenance of a biochemically lean environment likely characterize the mandible's relative protection and structural resilience in the face of systemic estrogen deficiency. |
| - | - | The observational design of this study, conducted at a single endpoint, focuses on establishing associations rather than defining direct causative mechanisms. These findings provide a foundational framework for future longitudinal studies to track the temporal progression of such metabolic shifts. Subsequent mechanistic research, incorporating direct assessments of lipid metabolism and enzymatic activity, will be essential to elucidate whether these lipid alterations actively contribute to bone pathology or function as descriptive biomarkers of systemic estrogen deficiency. |

### • Reviewer #2, Comment 2

A second point concerns the asymmetry in the experimental design. The mandible is analyzed in both bone marrow and mineralized tissue, whereas the tibia is analyzed only in mineralized tissue. This difference limits direct comparability between skeletal sites, particularly for compartment-specific effects, and may influence the interpretation of site-specific lipid patterns. While the manuscript notes that tibial marrow samples were reserved for a separate investigation, it would be helpful to further clarify the rationale for this choice and to discuss how the absence of tibial BM data may impact the conclusions.

***Note: this answer is duplicated for Reviewer #1, Comment 3 and Reviewer #2, Comment 2.***

The reviewer raises a valid point regarding transparency; we agree that clarifying the sampling rationale improves the manuscript's context.

The bone marrow of tibia was not available. This research was indeed developed in coordination with an ancillary study, which provided the opportunity to specifically examine the mandibular lipid profile in OVX condition compared to SHAM condition. We chose to focus on the mandible not only due to this availability but also because of its unique anatomical features; specifically, the absence of a medullary canal makes its biological organization distinct from long bones like the tibia.

Regarding the comparison with the tibia, we utilized the available mineralized tissue (MT) fraction as a secondary objective to build upon our previous laboratory data on long bone [3]. While we acknowledge that this comparison is limited to the MT compartment, we believe these findings offer a valuable complementary perspective.

In accordance with the reviewer's suggestion, we have revised the Objectives and Discussion sections to more explicitly describe the sampling rationale and the resulting focus of our analysis.

| Line (old) | Original | Modification |
| --- | --- | --- |
| 51-55 | In female Sprague-Dawley rats, we investigated whether estrogen deficiency induced by ovariectomy, compared to sham-operated (SHAM) controls and tibial sites, affects the mandibular fatty acid profile, including individual proportions and ratios in both MT and BM. A secondary question addressed the site effect, namely whether mandibular and tibial lipid profiles differ under both SHAM and OVX conditions. | In female Sprague-Dawley rats, we investigated whether estrogen deficiency induced by ovariectomy, compared to sham-operated (SHAM) controls ~~and tibial sites~~, affects the mandibular fatty acid profile, including individual proportions and ratios in both MT and BM. A secondary question addressed the site effect, namely whether mandibular and tibial lipid profiles differ under both SHAM and OVX conditions in MT. |
| 130-131 | Tibial BM samples were reserved for a separate investigation and were not analyzed in the present study. | The tibial BM samples were processed to answer other scientific questions and therefore could not be used in the present study. |
| 434 | - | A primary limitation of this study is the absence of tibial BM analysis, which resulted in an asymmetric experimental design. This was necessitated by the unavailability of the tibial BM, used in a separate investigation.  The lipid profiles observed in the tibial MT align with our previous findings regarding femoral MT in OVX rats [3]. In both long bones, MT demonstrated a characteristic increase in FA concentrations and MUFA enrichment, coupled with elevated Δ9 desaturase indices and diminished levels of 20:4 n−6 and 22:6 n−3. These parallel findings support the hypothesis that the tibial BM response mirrors the femoral BM profile, which was previously characterized by marked FA accumulation, increased Δ9 desaturase indices, and a decline in 20:4 n−6 and 22:6 n−3, as well.  While the mandibular BM followed a comparable metabolic trajectory, characterized by significant FA accumulation and similar shifts in desaturase activity, less pronounced than those observed in the femur [3]. Ultimately, although the mandible displays directional shifts similar to the femur and tibia, its distinct baseline and final lipid levels underscore a site-specific physiological response that distinguishes the mandible from the long bones. |

### • Reviewer #2, Comment 3

The microCT methodology also needs some clarification. The mandible and tibia were scanned using different acquisition settings, including markedly different voxel sizes and conditions. These differences may affect the comparability of measurements across skeletal sites. While the use of distinct settings is briefly justified by technical constraints, it would be helpful for the authors to discuss how this may influence the interpretation of site-specific differences, particularly the absence of detectable changes in the mandible.

The reviewer’s point concerning microCT acquisition settings is highly relevant. We would like to clarify that inter-site statistical comparisons were not performed. Given the anatomical differences between the tibia (long bone) and the mandible (irregular bone), they were treated as distinct entities. Our objective was not to compare absolute structural values between sites, but to parallel the relative impact of ovariectomy (SHAM vs. OVX) within each skeletal site. Within each site, the SHAM and OVX groups were scanned using strictly identical acquisition parameters and voxel sizes. This ensures that the intra-site comparisons and the resulting conclusions regarding mandibular resistance are mathematically and statistically sound.

Regarding the specific voxel sizes used (20 µm for the mandible and 4 µm for the tibia), we acknowledge that resolution can influence absolute morphometric values. According to Christiansen et al. [6], increasing voxel size typically leads to an overestimation of trabecular thickness (Tb.Th) and trabecular separation (Tb.Sp) and an underestimation of trabecular number (Tb.N). However, since this bias is applied equally to both SHAM and OVX groups within the mandible, it does not invalidate the observed absence of significant changes. Both 20 µm and 4 µm remain appropriate resolutions for characterizing the primary parameters discussed in this rat model.

### • Reviewer #2, Comment 4

Finally, some methodological aspects would benefit from further clarification. The separation of bone marrow and mineralized tissue is central to the study, and while a published protocol is followed, no direct validation of compartment purity is presented. It would be helpful to indicate how potential residual contamination was assessed or controlled.

Recognizing the technical importance of compartment purity, as highlighted by the reviewer, we have detailed our adherence to the cleaning protocol, as the separation of bone marrow from mineralized tissue is indeed a technical challenge. To mitigate the risk of residual marrow fat contamination, we strictly followed the rigorous cleaning protocol established by During (2017) [7].

Specifically, the BM was harvested through two successive steps using water-alcoholic solutions under agitation to ensure maximal removal of the bone marrow. The remaining bone fragments underwent two additional saline washes) to eliminate any ethanol residuals and BM-derived lipids.

The effectiveness of this control was assessed by During (2017) by analyzing the lipid loss during these final cleaning steps, which accounted for 7.5 +/- 2.5 % of the total recovered lipids. The lipid profile of these washings was found to be 91.3 % neutral lipids (predominantly triglycerides), a composition nearly identical to that of the BM itself. This indicated that the washing steps successfully captured residual marrow fat before the MT was cryomilled.

| Line (old) | Original | Modification |
| --- | --- | --- |
| 127-129 | Mineralized segments were fragmented and washed to minimize BM contamination, then cryomilled at −196°C into fine powder to preserve fatty acid integrity. | For the tibia, the medullary canal was initially flushed twice with a water-alcoholic (70% ethanol) solution; this step was omitted for the mandible due to the absence of a medullary cavity. Following fragmentation of both the tibia and mandible, the BM was harvested through two successive washes with the same solution under constant agitation to ensure maximal recovery.  To eliminate any residual BM lipids, the remaining bone fragments underwent two additional saline washes with 0.9% NaCl at 40°C to eliminate any residual bone marrow lipids. Tissues were then cryomilled at −196°C into fine powder to preserve fatty acid integrity. |

### • Reviewer #2, Comment 5

In addition, the use of desaturation indices as proxies for enzymatic activity is based on indirect assumptions and would benefit from further justification, particularly in the context of bone tissue. How do the authors support the interpretation of these ratios as reflecting underlying metabolic activity?

We appreciate the opportunity to further justify the use of desaturation indices as proxies for metabolic activity within bone tissue. The use of product-to-precursor ratios is a well-established methodology in lipid research for estimation of Δ9 desaturase (SCD-1) activity in vivo [8,9]. The SCD1 presence in the bone marrow has been demonstrated in the rat [10]. There is evidence of association between that enzyme activity and different metabolic pathways of lipids, namely de novo lipogenesis and beta-oxydation [8,11].

Moreover, clinical evidence supports the relevance of these indices in bone pathology. The serum 16:1/16:0 ratio has been linked to a higher risk of fractures in men [12], as well as a higher 18:1/18:0 ratio was observed in bone marrow fluid from post-menopausal women with hip fractures, compared to those without fracture [13]. These findings suggest that Δ9 desaturase indices provide valuable insight into the biological changes associated with bone fragility and osteoporosis.

The manuscript has been modified accordingly in the Discussion section to reflect this justification.

| Line (old) | Original | Modification |
| --- | --- | --- |
| 331-333 | By combining an apparently restricted Δ9 desaturase activity with an enrichment in pro-resolving PUFAs, the mandible maintains a biochemical environment inherently less susceptible to adverse remodeling. | By combining ~~an apparently~~ restricted Δ9 desaturase activity indices with an enrichment in pro-resolving PUFAs, the mandible maintains a biochemical environment inherently less susceptible to adverse remodeling. |
| 369-372 | The preservation of high 20:4 n−6 and 22:6 n−3, and lower Δ9 desaturase activity, characterize the mandibular response. | The preservation of high 20:4 n−6 and 22:6 n−3, and lower Δ9 desaturase indices, characterize the mandibular response. |
| - | - | To estimate Δ9 desaturase (SCD-1) activity, we used product-to-precursor ratios (16:1/16:0 and 18:1/18:0), which are standard proxies in lipid research [8,9]. The presence of SCD-1 protein in the rat BM has been shown [10], and its enzymatic activity is linked to metabolic pathways including de novo lipogenesis and β-oxidation in adipocytes [8,11]. Given that the 16:1/16:0 ratio was associated with fracture risk in men [12], as well as a higher 18:1/18:0 ratio was observed in bone marrow fluid from post-menopausal women with hip fractures, compared to those without fracture [13]. These indices might provide valuable insight into the lipid profiles of resilience across different skeletal sites following ovariectomy. |

### • Conclusion

***By refining our terminology to focus on associations rather than direct mechanisms and providing deeper methodological justification for our analytical choices, we believe the manuscript now offers a more transparent and scientifically grounded interpretation of the mandibular lipid profile.***

# Additional remarks

We found a discrepancy in the Concentration data. After careful reconsideration, it lowers the original concentrations in the mandible. However, it does not change any conclusion, and does not affect the fatty acid relative proportions (computations were not linked).

All subsequent modification are shown here:

| Line (old) | Original | Modification |
| --- | --- | --- |
| 206-207 | Table 2 (original values) | Table 2 (reconsidered values) |
| 231 | Figure 2 | Figure 2 (updated) |
| 245 | Figure 3 | Figure 3 (updated) |
| 214-215 | Across compartments, OVX rats showed higher total FA concentrations, with increases in mandibular BM (p = 0.01), tibial MT (p = 0.004), and mandibular MT (p = 0.01). | Across compartments, OVX rats showed higher total FA concentrations, with increases in mandibular BM (p = 0.01), tibial MT (p = 0.01), and mandibular MT (p = 0.01). |
| 217-218 | In the SHAM group, total FA concentrations were comparable between mandibular and tibial MT (p = 0.20, Figure 2). | In the SHAM group, total FA concentrations were comparable between mandibular and tibial MT (p = 0.14, Figure 2). |
| 240-242 | In the tibia, the MT from the OVX group showed a large lipid increase, nearly doubling compared to SHAM group (+91%). | In the tibia, the MT from the OVX group showed a large lipid increase~~, nearly doubling~~ compared to SHAM group (+69%). |
| 335-337 | In the tibia, MT total FA content nearly doubled (+91%), reaching 10,500 nmol/g, whereas the mandible showed a more modest increase (+35% to 6,500 nmol/g in MT, +68% to 8,000 nmol/g in BM). | In the tibia, MT total FA increased (+69%), reaching 6,000 nmol/g, whereas the mandible showed a milder increase in MT (+35% to 4,700 nmol/g in MT) and a similar increase in BM (+68% to 6,000 nmol/g in BM). |
| 432-435 | Quantitatively, the total fatty acids were higher in tibial mineralized tissue in OVX groups (91%) than in mandibular compartments (35% in MT and 68% in BM), underscoring the mandible’s reduced lipid accumulation under estrogen deficiency. | Quantitatively, the total fatty acids were higher in ~~tibial~~ mineralized tissue in OVX groups, with a stronger increase in the tibia (+69%) than in the mandible (+35%), underscoring the mandible’s reduced lipid accumulation under estrogen deficiency within this compartment. |

# Bibliography

1. Thompson DD, Simmons HA, Pirie CM, Ke HZ. FDA guidelines and animal models for osteoporosis. Bone. 1995;17(4 Suppl):125S-133S. DOI: 10.1016/8756-3282(95)00285-l

2. Johnston BD, Ward WE. The Ovariectomized Rat as a Model for Studying Alveolar Bone Loss in Postmenopausal Women. Biomed Res Int. 2015;2015:e635023. DOI: 10.1155/2015/635023

3. During A, Coutel X, Bertheaume N, Penel G, Olejnik C. Long term ovariectomy-induced osteoporosis is associated with high stearoyl-CoA desaturase indexes in rat femur. Calcif Tissue Int. 2020;106(3):315‑24. DOI: 10.1007/s00223-019-00637-7

4. Kasonga AE, Deepak V, Kruger MC, Coetzee M. Arachidonic Acid and Docosahexaenoic Acid Suppress Osteoclast Formation and Activity in Human CD14+ Monocytes, In vitro. PLOS ONE. 2015;10(4):e0125145. DOI: 10.1371/journal.pone.0125145

5. Sessler AM, Kaur N, Palta JP, Ntambi JM. Regulation of stearoyl-CoA desaturase 1 mRNA stability by polyunsaturated fatty acids in 3T3-L1 adipocytes. J Biol Chem. 1996;271(47):29854‑8. DOI: 10.1074/jbc.271.47.29854

6. Christiansen BA. Effect of micro-computed tomography voxel size and segmentation method on trabecular bone microstructure measures in mice. Bone Rep. 2016;5:136‑40. DOI: 10.1016/j.bonr.2016.05.006

7. During A. Lipid determination in bone marrow and mineralized bone tissue: From sample preparation to improved high-performance thin-layer and liquid chromatographic approaches. J Chromatogr A. 2017;1515:232‑44. DOI: 10.1016/j.chroma.2017.08.004

8. Hodson L, Fielding BA. Stearoyl-CoA desaturase: rogue or innocent bystander? Prog Lipid Res. 2013;52(1):15‑42. DOI: 10.1016/j.plipres.2012.08.002

9. Delattre J, Bertheaume N, Olejnik C, During A. Changes in local lipids are correlated with bone microarchitecture alterations during aging and ovariectomy-induced osteoporosis in rat femur: focus on the Δ9-desaturase 18:1/18:0 index and arachidonic acid level. Lipids Health Dis. 2026;25(45). DOI: 10.1186/s12944-026-02858-w

10. Mori H, Dugan CE, Nishii A, Benchamana A, Li Z, Cadenhead TS, et al. The molecular and metabolic program by which white adipocytes adapt to cool physiologic temperatures. PLoS Biol. 2021;19(5):e3000988. DOI: 10.1371/journal.pbio.3000988

11. Kim E, Lee JH, Ntambi JM, Hyun CK. Inhibition of stearoyl-CoA desaturase1 activates AMPK and exhibits beneficial lipid metabolic effects in vitro. Eur J Pharmacol. 2011;672(1‑3):38‑44. DOI: 10.1016/j.ejphar.2011.09.172

12. Melhus H, Risérus U, Warensjö E, Wernroth L, Jensevik K, Berglund L, et al. A high activity index of stearoyl-CoA desaturase is associated with increased risk of fracture in men. Osteoporos Int. 2008;19(7):929‑34. DOI: 10.1007/s00198-007-0521-y

13. Miranda M, Pino AM, Fuenzalida K, Rosen CJ, Seitz G, Rodríguez JP. Characterization of fatty acid composition in bone marrow fluid from postmenopausal women: modification after hip fracture. J Cell Biochem. 2016;117(10):2370‑6. DOI: 10.1002/jcb.25534
